# MASP-1 Induces a Unique Cytokine Pattern in Endothelial Cells: A Novel Link between Complement System and Neutrophil Granulocytes

**DOI:** 10.1371/journal.pone.0087104

**Published:** 2014-01-29

**Authors:** Péter K. Jani, Erika Kajdácsi, Márton Megyeri, József Dobó, Zoltán Doleschall, Krisztina Futosi, Csaba I. Tímár, Attila Mócsai, Veronika Makó, Péter Gál, László Cervenak

**Affiliations:** 1 3rd Department of Internal Medicine, Semmelweis University, Budapest, Hungary; 2 Institute of Enzymology, Research Centre for Natural Sciences, Hungarian Academy of Sciences, Budapest, Hungary; 3 Department of Pathogenetics, National Institute of Oncology, Budapest, Hungary; 4 Department of Physiology, Semmelweis University School of Medicine, Budapest, Hungary; Federal Institute for Vaccines and Biomedicines, Germany

## Abstract

Microbial infection urges prompt intervention by the immune system. The complement cascade and neutrophil granulocytes are the predominant contributors to this immediate anti-microbial action. We have previously shown that mannan-binding lectin-associated serine protease-1 (MASP-1), the most abundant enzyme of the complement lectin pathway, can induce p38-MAPK activation, NFkappaB signaling, and Ca^2+^-mobilization in endothelial cells. Since neutrophil chemotaxis and transmigration depends on endothelial cell activation, we aimed to explore whether recombinant MASP-1 (rMASP-1) is able to induce cytokine production and subsequent neutrophil chemotaxis in human umbilical vein endothelial cells (HUVEC). We found that HUVECs activated by rMASP-1 secreted IL-6 and IL-8, but not IL-1alpha, IL-1ra, TNFalpha and MCP-1. rMASP-1 induced dose-dependent IL-6 and IL-8 production with different kinetics. rMASP-1 triggered IL-6 and IL-8 production was regulated predominantly by the p38-MAPK pathway. Moreover, the supernatant of rMASP-1-stimulated HUVECs activated the chemotaxis of neutrophil granulocytes as an integrated effect of cytokine production. Our results implicate that besides initializing the complement lectin pathway, MASP-1 may activate neutrophils indirectly, via the endothelial cells, which link these effective antimicrobial host defense mechanisms.

## Introduction

The immune system responds to various pathogens through different sets of immune mechanisms. The predominant, and most effective initial mechanisms for eliminating bacterial or fungal infection are the complement system and neutrophil granulocytes.

The complement system is part of the innate immune system. It can recognize, identify, and eliminate invading pathogens and altered host cells. The complement system can be activated through three different routes: the classical, the lectin, and the alternative pathways. The pattern-recognition molecules of the lectin pathway – mannan-binding lectin (MBL), collectin 11 (CL-K1), and the three ficolins (H-, L-, and M-ficolin) – circulate in the blood in complexes with MBL-associated serine proteases (MASP-1, 2 and 3), and mannose-binding lectin associated proteins (MAp19 and MAp44). When MBL, CL-K1, or ficolins recognize a microorganism, the activation of MASP-1, as a promiscuous protease [1], leads to several distinct outcomes: 1) activation of the complement system via cleavage of MASP-2 [2], [3], 2) cleavage of kininogen and the release of bradykinin [4], 3) cleavage of fibrinogen and factor XIII (transglutaminase) [5], 4) activation of endothelial cells via protease activated receptor 4 (PAR-4, a member of GPCR family) signaling, as we have previously described [6]. Briefly, we showed that rMASP-1 can cleave PAR-1, 2, and 4 with different efficacy, and PAR-4 activation leads to Ca^2+^-signaling, the nuclear translocation of NFkappaB, and the phosphorylation of p38-MAPK. The cleavage of endothelial PAR-1 and PAR-4 by thrombin causes changes in endothelial cell morphology, as well as in the release of vasoactive substances and cytokines [7]. In general, cytokine-generation during the inflammatory response requires the involvement of the p38-MAPK, JNK, NFkappaB or cAMP responding element-binding protein (CREB) signaling pathways [8]–[12].

The endothelium can generate anti-inflammatory cytokines such as IL-1ra (receptor antagonist), as well as pro-inflammatory cytokines (e.g. IL-1alpha, IL-6, IL-8, MCP-1 and TNFalpha) in response to various stimuli [8], [10]–[13]. TNFalpha, IL-1alpha, and IL-6 are the most important mediators of the acute phase response, and of fever. Furthermore, TNFalpha can regulate the levels of the proteins required for antigen presentation. IL-1alpha is a regulator of Th1/Th2 balance, whereas IL-6 is a potent survival factor of plasma cells, and participates in IgA class switching. IL-1ra is a natural inhibitor of the pro-inflammatory IL-1beta cytokine [11]. IL-8 and MCP-1 (monocyte chemoattractant protein) are chemokines, which control the migration of selected leukocyte subsets into inflamed tissues. IL-8 and MCP-1 are chemoattractants for neutrophil granulocytes and monocytes, respectively [14]. IL-6, IL-8 and MCP-1 are secreted with a low constitutive rate by endothelial cells; however, they can also be stored in different granular structures (Weibel-Palade bodies and type-2 chemokine-containing organelles) [15]. Upon pro-inflammatory stimuli, both rapid degranulation, and *de novo* protein synthesis may result in the elevated secretion of these cytokines. Although IL-6, IL-8, and MCP-1 are regulated similarly in most cases, there are also dissimilarities in their secretion. One major difference is the lacking P-CREB binding site in the promoter region of MCP-1, but notwithstanding, the expression of IL-6 and IL-8 can be driven by CREB [9], [16]–[18]. Differential chemokine production, together with the adhesion molecule pattern, can be the most important regulators of leukocyte trafficking driven by the endothelium. The effector function of neutrophil granulocytes (polymorphonuclear cells, PMN) is a multi-step process. Chemotaxis precedes transmigration through endothelial cell junctions, the production of reactive oxygen species, and microbial killing.

In this study, we described that a unique cytokine profile, produced by rMASP-1-stimulated HUVECs, is able to induce chemotaxis of neutrophil granulocytes, as a novel link between the complement system and the endothelial cell-mediated regulation of the neutrophil response.

## Materials and Methods

### Reagents

Recombinant human MASP-1 catalytic fragments (CCP1-CCP2-SP, abbreviated as rMASP-1) were expressed in *E. coli* and prepared according to Dobó *et al.* [1], and Ambrus *et al.* [19], respectively. The recombinant enzyme preparations were free of bacterial contaminations and the effects of rMASP-1 could be inhibited by C1-Inhibitor as described by Megyeri *et al.* [6] We used lyophilized plasma C1-Inhibitor (Berinert P; ZLB Behring), the natural inhibitor of MASP-1. The specific JNK II inhibitor (SP600125), the p38-MAPK inhibitor (SB203580), the PI3-Kinase inhibitor (Wortmannin), the NFkappaB inhibitor (Bay 11-7082), and the MEK-1/2 inhibitor (U0126) were purchased from Calbiochem (Merck KGaA, Darmstadt, Germany). Other reagents were purchased from Sigma-Aldrich, unless otherwise stated. All inhibitors were tested and optimized in preliminary experiments (data not shown).

### Preparation and culture of human umbilical vein endothelial cells (HUVECs)

Cells were harvested from fresh umbilical cords obtained during normal delivery of healthy neonates (according to Helsinki Protocol, Semmelweis University Institutional Review Board specifically approved this study, (permission number: TUKEB64/2008), and all participants provided their written informed consent to participate in this study), by collagenase digestion as described by Oroszlan *et al* [20]. HUVECs were kept in gelatin-precoated flasks in AIM-V medium (Invitrogen) completed with 1% FCS, 2 ng/ml human recombinant epidermal growth factor (R&D Systems), 250 pg/ml human recombinant β-endothelial cell growth factor (BioSource/Invitrogen), and 7.5 U/ml heparin, hereinafter referred to as Comp-AIM-V. Each experiment was performed on at least three independent primary HUVEC cultures from different individuals.

### Measurement of CREB phosphorylation by immunofluorescence microscopy

HUVECs were treated with/without 2 µM of rMASP-1, in 100 µl Comp-AIM-V for 25 minutes, fixed and stained with rabbit-anti-human phospho-CREB (P-CREB, 1∶200, Cell Signaling Technology Inc.) antibody followed by Alexa568 conjugated goat-anti-rabbit IgG (1∶500) and Hoechst 33342 (1∶50000, Molecular Probes/Invitrogen) as described previously [8]. All analyses were performed using the original, unmodified images; for visualization, the pictures were modified according to a standardized procedure, using Adobe Photoshop CS, without gamma-correction.

### Analysis of CREB and JNK phosphorylation by Western blotting

Confluent cell cultures in 25 cm^2^ flasks were treated with rMASP-1, thrombin, TNFalpha or IL-1beta for 25 minutes. After washing with ice-cold PBS, cells were lysed in buffer containing 30 mM Hepes, pH 7.4, 100 mM NaCl, 1 mM EDTA, 20 mM NaF, 1 mM PMSF, 1 mM Na_3_VO_4_ and 2% protease inhibitor cocktail (BD Bioscience). For the P-CREB analysis, the buffer described above was supplemented with 1% Triton-X100. Samples were separated by 12% SDS-polyacrylamide gel electrophoresis (SDS-PAGE), transferred onto PVDF membranes, and probed as we described previously [8]. For CREB and phospho-CREB staining, we used the same antibodies as for microscopy.

### mRNA analysis

Variously treated HUVECs were lysed and stored in TRI® reagent. Total RNA purification, reverse transcription and LightCycler® analysis were performed as previously described by Megyeri *et al* [6]. The primers (Table 1) were designed from NCBI database, and produced by Bio Basic Canada Inc. GAPDH and β-actin gene-specific primers were synthesized according to the published cDNA sequences. The purity and the size of the PCR products were checked by melting curve analysis and agarose gel-electrophoresis.

**Table 1:** The primers used in the qPCR reactions.

**interleukin-1 receptor antagonist**	*forward:*	5′-gatacttgcaaggaccaaatgtc-3′
	*reverse:*	5′-gtctcatcaccagacttgacaca-3′
**interleukin-1a**	*forward:*	5′-gcttcctgagcaatgtgaaatac-3′
	*reverse:*	5′-tgacttataagcacccatgtcaa-3′
**interleukin-6**	*forward:*	5′-ctgcaggacatgacaactcatc-3′
	*reverse:*	5′-atctgaggtgcccatgctac-3’
**interleukin-8**	*forward:*	5′-tcctgatttctgcagctctgt-3′
	*reverse:*	5′-tgtggtccactctcaatcactc-3′
**monocyte chemoattractant protein-1**	*forward:*	5′-caccaataggaagatctcagtgc-3′
	*reverse:*	5′-tgagtgttcaagtcttcggagtt-3′
**tumor necrosis factor-α**	*forward:*	5′-tgtagcccatgttgtagcaaac-3′
	*reverse:*	5′-gacctgggagtagatgaggtacag-3′

### Measurement of cytokine production by xMAP technology

Confluent layers (10^5^ cell/well) of HUVECs were cultured onto 96-well plates overnight in 100 µl Comp-AIM-V medium in the presence of rMASP-1, LPS, IL-1beta and TNFalpha. The optimal concentration of activators was pre-determined in our previous studies [6], [8], [21]. Supernatants were diluted 1∶30 (for MCP-1 and IL-8) or 1∶2 (for IL-1alpha, IL-1ra, IL-6 and TNFalpha). The human Fluorokine MAP Base Kit (PanelA, R&D Systems) was used according to the manufacturer's instructions, and measured in a Luminex 100 reader (Luminex Corporation, Austin, Texas).

### Measurement of cytokine production by sandwich ELISA

Confluent layers (10^5^ cell/well) of HUVECs were cultured in 96-well plates for 24 hours in 100 µl Comp-AIM-V medium in the presence of rMASP-1 or other endothelial cell activating factors. IL-1alpha, IL-1ra, IL-6, IL-8, MCP-1, and TNFalpha were measured by sandwich ELISA kits according to the manufacturer's protocol (R&D Systems). We also analyzed the production of IL-6 and IL-8 at 1, 3, 6, 10, and 24 hours.

### Analysis of IL-6, IL-8 production in presence of pathway inhibitors

Confluent layers of HUVECs were pre-incubated for 30 minutes with the following pathway inhibitors: SP600125, 25 µM – JNK; SB203580, 2 µM - p38-MAPK; Wortmannin, 100 nM – PI3-K; Bay-11 7082, 5 µM - NFkappaB and U0126, 1 µM - MEK1/2. The effective, non-toxic dose of different pathway inhibitors was determined in our preliminary experiments, and was similar to the literature data. For rMASP-1 treatment, we used the purified C1-Inhibitor – an endogenous, irreversible inhibitor of MASP-1 — as negative control. rMASP-1 was pre-incubated with C1-Inhibitor for 30 minutes before treatment. The IL-6 and IL-8 content of the supernatant were determined at 3 and 24 hours as described beforehand.

### Measurement of rMASP-1 concentration and rMASP-1/C1-Inhibitor disintegration

We measured the activity of rMASP-1 using LPAPR-AMC fluorescent substrate as previously described [6]. Concentration of rMASP-1 was calculated from the slopes of the kinetic curves. The detection limit of rMASP-1 was 0.5 nM.

In order to assess residual rMASP-1 activity during the assays on HUVECs, 2 µM rMASP-1 was pre-incubated with or without equimolar concentration of C1-Inhibitor. Small aliquots were removed at 0; 0,5; 1; 2; 3; 6; 8 and 24 hours, and tested for specific rMASP-1 activity.

### Neutrophil preparation

Human neutrophils were isolated from venous blood of healthy volunteers by Ficoll gradient centrifugation, followed by hypotonic lysis of RBCs [22]. Cells were resuspended in Ca^2+^/Mg^2+^-free HBSS supplemented with 20 mM HEPES, pH 7.4, and used immediately. All experiments on human neutrophil granulocyte samples were approved by the institutional review board of Semmelweis University. All participants provided their written informed consent to participate in this study.

### Preparation of HUVEC supernatants for experiments with neutrophil granulocytes

To avoid any possible direct effects of rMASP-1 on neutrophil granulocytes, HUVECs were treated or not with 2 µM rMASP-1 for 30 minutes then the medium was replaced by rMASP-1 free HBSS containing 0.5 mM CaCl_2_ and 1 mM MgCl_2_ for additional 2,5 hours. Then the HBSS supernatants (MASP-SN - rMASP-1 treated HUVEC supernatant, or UNT-SN - untreated HUVEC supernatant) were collected and stored frozen (−80°C) until use.

### Superoxide production

To measure reactive oxygen species production, a lucigenin-based chemiluminescence method was used [23]. Briefly, the cells were activated with MASP-SN or UNT-SN for 3 hours. In other experiments, we used HUVEC supernatants as preconditioning stimuli for 20 minutes and then, the response to 100 µM phorbol 12-myristate 13-acetate (PMA) or 1 µM N-formyl-methionyl-leucyl-phenylalanine (fMLP) was recorded.

### Neutrophil chemotaxis assay

Isolated neutrophils were diluted in assay medium, supplemented with 0.5 mM CaCl_2_ and 1 mM MgCl_2_, and then, placed into Transwell inserts with FCS-coated 3 μm polycarbonate filters (Corning) essentially as described previously [22], [24]. The Transwell inserts were placed into 24-well plates containing MASP-SN, UNT-SN or 2 ng/ml IL-8 (Peprotech). Neutrophil migration over 60 minutes was quantified using an acid phosphatase assay [24].

### Statistical analysis

Data were compared using Student's *t*-test or one-way ANOVA with Tukey's post-test (GraphPad Prism 5.01 software, GraphPad, http://www.graphpad.com/). A *p*-value less than 0.05 was considered significant. Data are presented as means ± SEM unless otherwise stated.

## Results

### Activation of CREB and JNK by rMASP-1

Previously, we reported that rMASP-1 could activate p38-MAPK, NFkappaB, and Ca-mobilization in HUVECs. We also demonstrated that the cleavage of PARs might be the initiator of rMASP-1 signaling [6]. Since PARs also utilize – through G-protein coupled signaling – other pathways to induce pro-inflammatory cytokines, first we examined CREB and JNK activation by rMASP-1.

**Figure 1:**
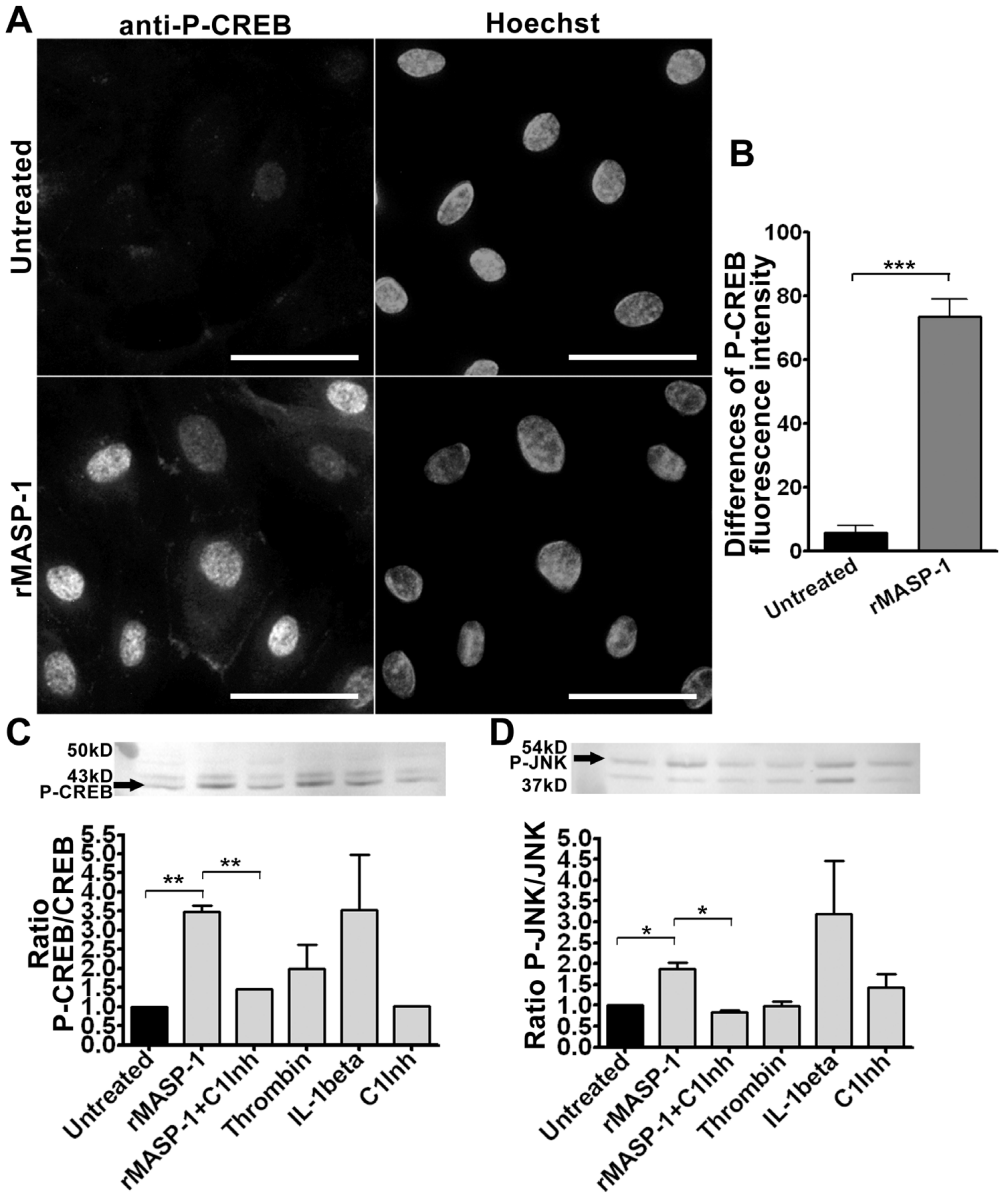
rMASP-1 treatment of HUVEC activates CREB and JNK signaling pathways. The cells were treated or not treated with 2 µM rMASP-1 for 25 minutes and then, fixed with ice cold methanol-acetone (1∶1), labeled overnight at 4°C with 1∶200 diluted rabbit anti-human Phosopho-CREB antibody, and stained with goat anti-rabbit (1∶500) Alexa 568 (left column) and Hoechst nuclear staining (right column). The figure depicts one out of 5 similar, independent experiments. The bars represent 50 µm (**A**). The images were obtained by Olympus IX-81 inverted fluorescence microscope equipped with 40× UPLF objective (NA = 0,75), and an Olympus DP70 digital camera. The mean intensity of red fluorescence in the nuclear and perinuclear region was evaluated using the AnalySIS software. The mean (+/−SEM) differences between the indicated regions of 5 experiments are shown (**B**). Cells were seeded on 6-well plates and treated for 25 minutes. Cells were then lysed and Western-blots were performed. The membranes were probed for CREB and phospho-CREB (**C**) or JNK and phospho-JNK (**D**). Two representative phospho-CREB and phospho-JNK images are shown (linear intensity adjusted). The graphs were calculated from unadjusted values of phospho- and total protein ratios from 3 independent experiments. The significance of the differences among rMASP-1 and the other treatments is shown. *: p<0.05, **: p<0.01, ***: p<0.001, ns: non-significant.

We found that rMASP-1 treatment could activate CREB phosphorylation in HUVECs, compared with untreated cells (Figure 1/A,B). We confirmed our results by Western Blot analysis. The effect of rMASP-1 on CREB phosphorylation was comparable with that of thrombin and of IL-1beta, used as positive controls. The effect of rMASP-1 could be completely inhibited by co-incubation with the C1-Inhibitor, which by itself did not induce CREB phosphorylation (Figure 1/C). rMASP-1 was also able to induce JNK phosphorylation, and the C1-Inhibitor abolished this effect too. (Figure 1/D).

### Screening of cytokine production

**Figure 2:**
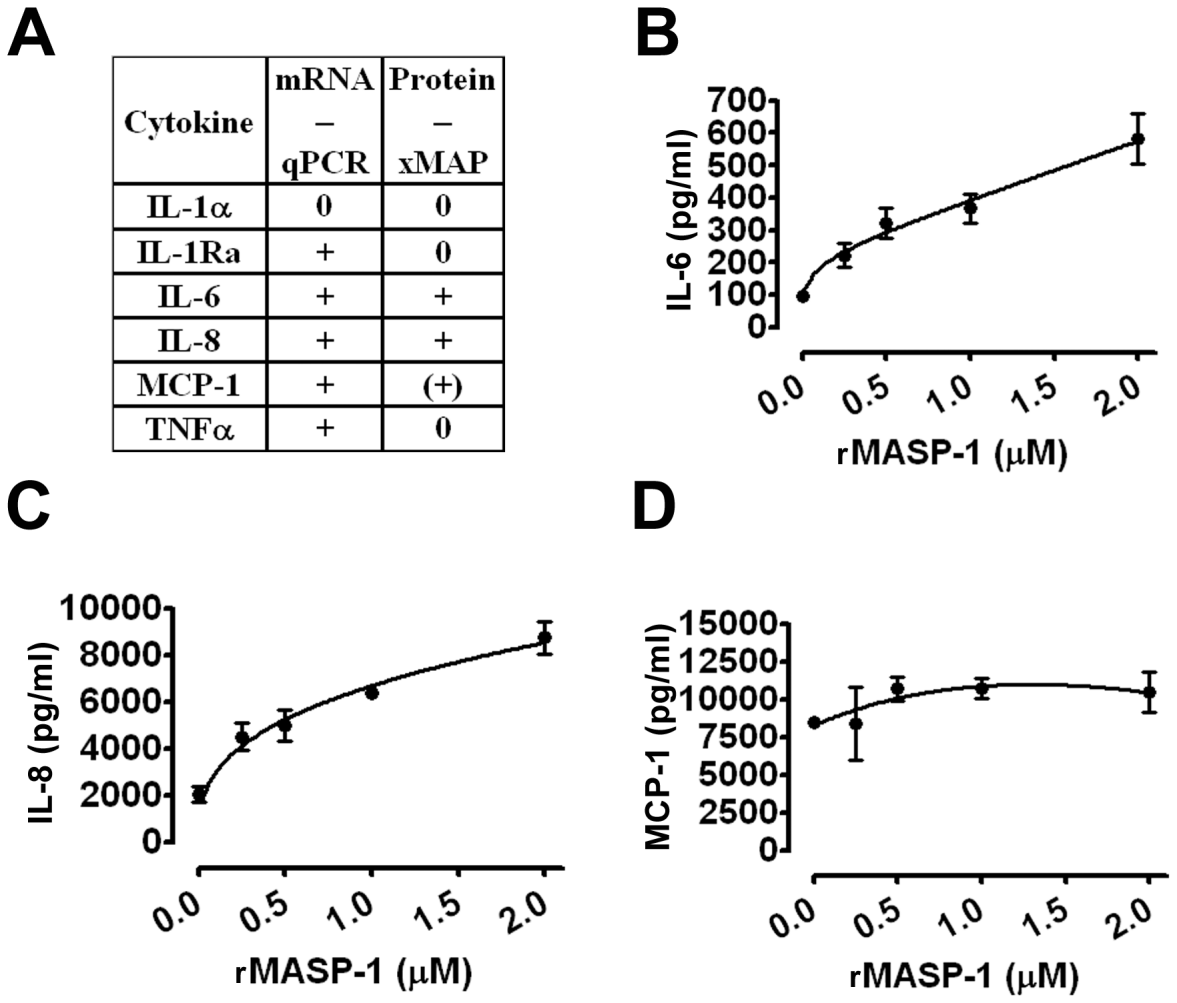
Screening for cytokine production induced by rMASP-1, and the dose dependence of IL-6, IL-8, and MCP-1. The cells were treated with 2 µM rMASP-1 for 6 hours (mRNA level) or 24 hours (protein level). Seven cytokines were assessed by qPCR and xMAP technology. (0: no effect, +: significant induction, (+): tendency for induction, not confirmed by ELISA.) (**A**) The cells were treated with 0, 0.25, 0.5, 1, 2 µM rMASP-1 for 24 hours, and then, the supernatants were collected. IL-6 (**B**), IL-8 (**C**), and MCP-1 (**D**) production were determined by sandwich ELISA kits according to the manufacturers' protocol. The values were calculated as the mean (+/−SEM) of three independent experiments.

Endothelial cells can produce several pro-, and anti-inflammatory cytokines. Therefore, we treated HUVECs with rMASP-1 for 6 hours and then, determined specific cytokine mRNA levels by qPCR. The level of IL-1ra, IL-6, IL-8, MCP-1 and TNFalpha mRNA was increased, whereas the amount of IL-1alpha mRNA did not change (Figure 2/A). In addition, we treated HUVECs with rMASP-1 for 24 hours, and measured the cytokine profile with xMAP technology. IL-6 and IL-8 production increased significantly. MCP-1 level was moderately, but not significantly higher than in untreated controls, whereas the production of IL-1alpha, TNFalpha, IL-1ra did not change (Figure 2/A).

### rMASP-1 dose dependence on IL-6 and IL-8 production

rMASP-1 treatment induced IL-6, and IL-8 production in a dose-dependent manner. In concentrations as low as 250 nM, rMASP-1 caused a significant increase in the levels of both cytokines (one-way ANOVA). Although endothelial cells secrete large amounts of MCP-1, rMASP-1 did not increase the production of the latter any further (Figure 2/B-D).

### Comparison of rMASP-1 with other endothelial cell activators

**Figure 3:**
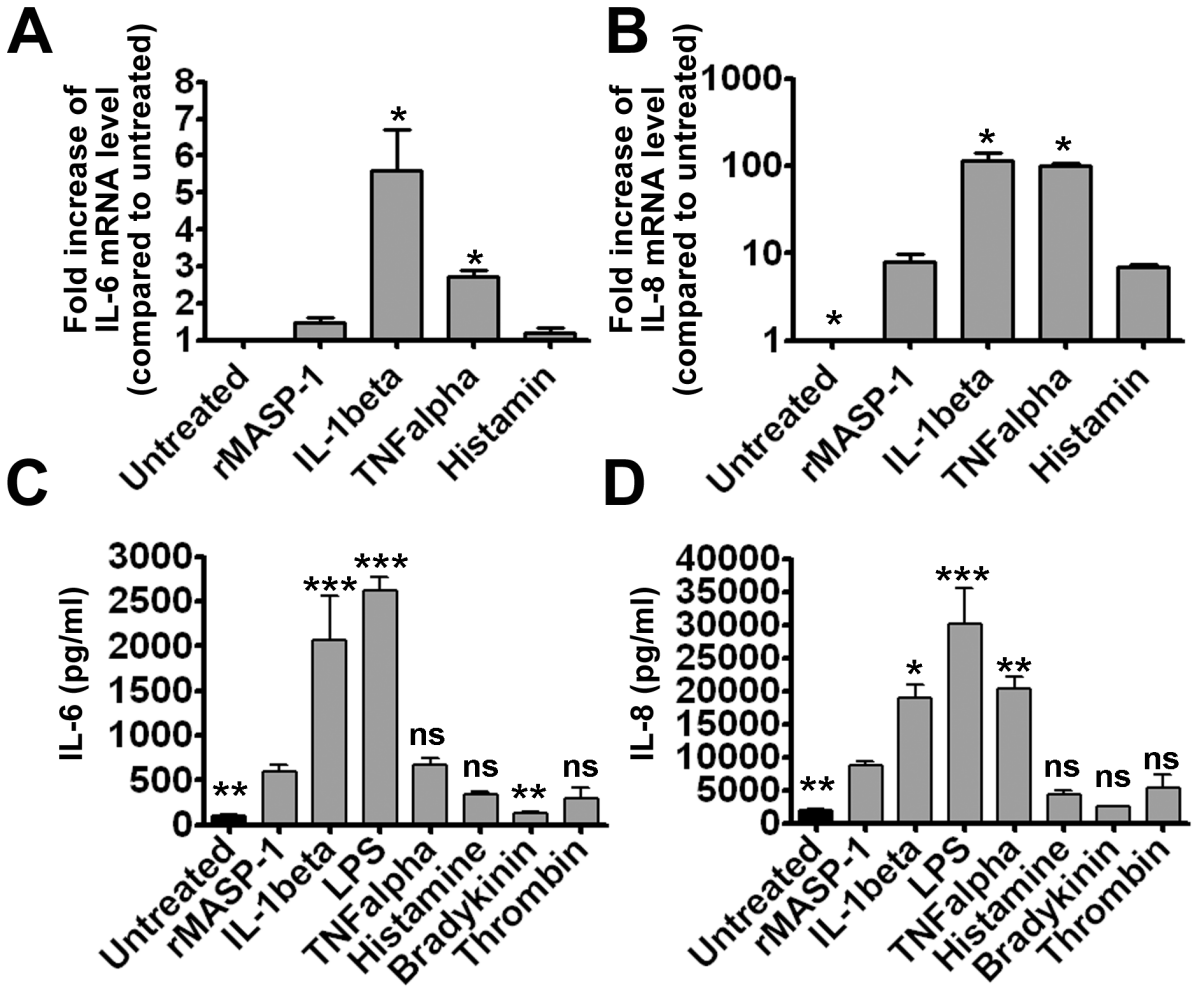
The comparison of rMASP-1 with other endothelial cell activators. HUVECs were treated with different cell activators (rMASP-1 2 µM, IL-1beta 1 ng/mL, TNFalpha 10 ng/mL, histamine 50 µM, LPS 100 ng/mL, bradykinin 20 µM, thrombin 300 nM) for 6 hours (**A, B**), or 24 hours (**C, D**). mRNA for qPCR (**A, B**), and the supernatants for ELISA (**C, D**) were collected and then, IL-6 (**A, C**) and IL-8 (**B, D**) expression was determined. All data are presented as the mean (+/−SEM) of three independent experiments. The significance of the differences among rMASP-1 and other treatments is shown. *: p<0.05, **: p<0.01, ***: p<0.001, ns: non-significant.

Several internal and external factors can trigger cytokine production in endothelial cells. A precise comparison of rMASP-1 with these factors (including fine dose- and time-dependence) was beyond the scope of our experiments. Nevertheless, we performed a simple analysis, where the effects of a single, pre-determined, optimized dose of histamine (50 µM), bradykinin (20 µM), thrombin (300 nM) [6], TNFalpha (10 ng/mL), IL-1beta (1 ng/mL), and LPS (100 ng/mL) [8] were compared with those of 2 µM rMASP-1. At 6 hours, rMASP-1 was a less potent inducer of IL-6 and IL-8 transcripts than IL-1beta or TNFalpha (Figure 3/A,B), but the effect of histamine was similar. At protein level, rMASP-1 induced IL-6 more effectively than bradykinin; its efficacy was similar to that of TNFalpha, histamine, and thrombin, but inferior to that of IL-1beta, or LPS (Figure 3/C). The effects of rMASP-1 and TNFalpha on IL-6 production were similar; however, TNFalpha produced more IL-8 than rMASP-1 (Figure 3/D).

### Kinetics of IL-6 and IL-8 production

**Figure 4:**
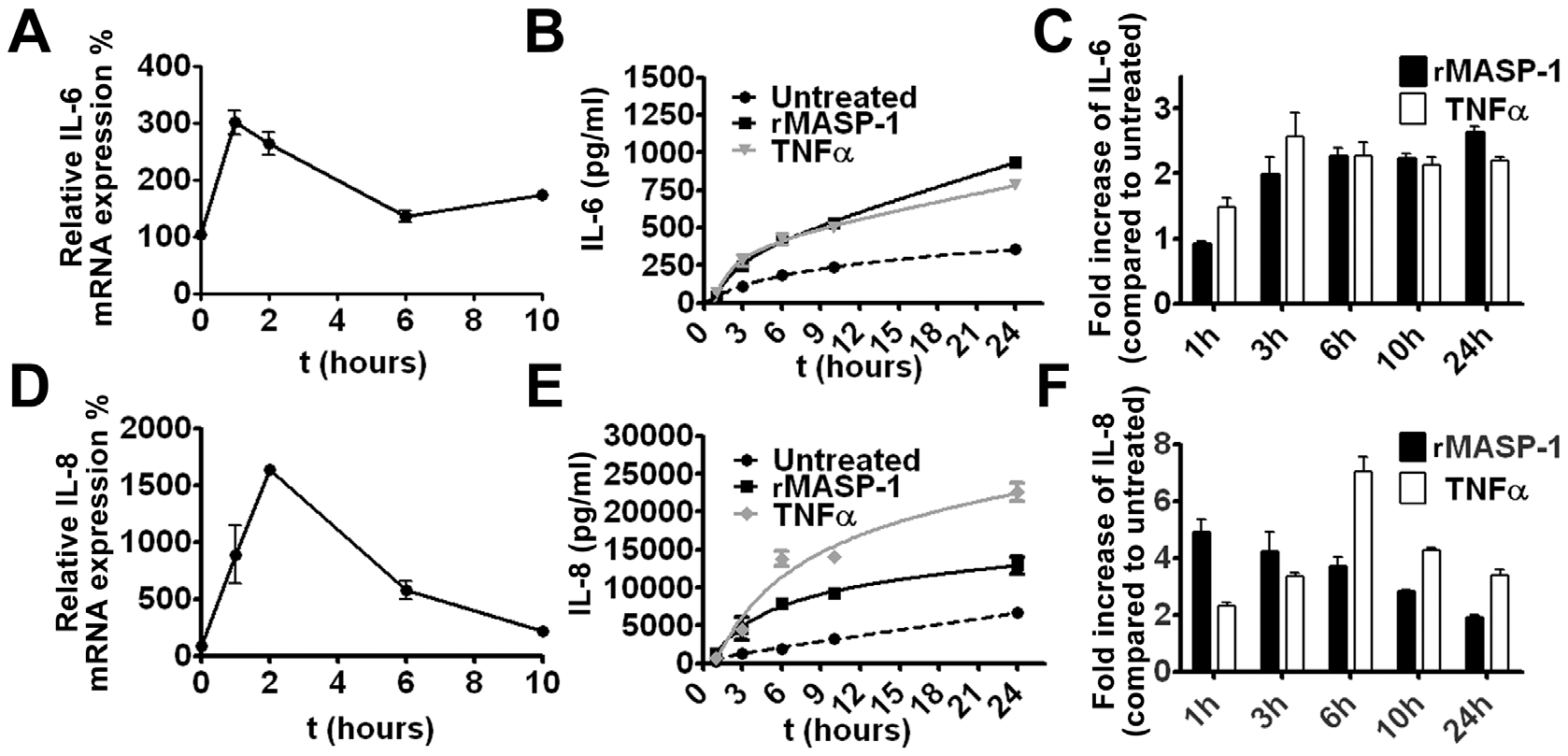
The kinetics of IL-6 and IL-8 production induced by rMASP-1. HUVECs were treated with 2 µM rMASP-1, or with 10 ng/mL TNFα (only at protein level) for different periods. The samples were analyzed for IL-6 (**A, B, C**) and IL-8 (**D, E, F**) by qPCR (**A, D**) and ELISA (**B, C, E, F**). qPCR results were plotted as percentage of non-treated control. The results of ELISA were calculated from the standard curve, and plotted as concentration values (**B, E**). To compare the effects of rMASP-1 and of TNFalpha, we calculated the magnitude of the increase of the values (treated/non-treated) at each time-point (**C, F**). All data are presented as the mean (+/−SEM) of three independent experiments.

IL-6 and IL-8 synthesis and secretion are regulated at multiple levels in endothelial cells. To determine whether the transcription or the exocytosis of pre-formed granules is the main contributor to their secretion, we performed kinetic measurements at mRNA and protein levels. HUVECs were treated with 2 µM rMASP-1, and samples for mRNA extraction were harvested at 1, 2, 6, and 10 hours. The mRNA level of IL-6 increased rapidly and peaked between 1 and 2 hours (Figure 4/A). The increase of the mRNA level of IL-8 was slower (the peak was around 2 hours), but greater (Figure 4/D). Then, HUVECs were treated with 2 µM rMASP-1, and supernatants for ELISA were collected at 1, 3, 6, 10, and 24 hours. TNFalpha was used as a positive control. Untreated HUVECs produced both IL-6 and IL-8 at a basal level. rMASP-1 and TNFalpha induced IL-6 with very similar kinetics (Figure 4/B,C). The kinetics of IL-8 production was, however, different in response to rMASP-1 and to TNFalpha. While rMASP-1 promptly induced IL-8 secretion (relative production compared to untreated cells peaked between 1 and 3 hours), the maximum effect of TNFalpha was at 6 hours (Figure 4/E,F).

### Unraveling the signaling pathways that contribute to IL-6 and IL-8 production induced by rMASP-1

**Figure 5:**
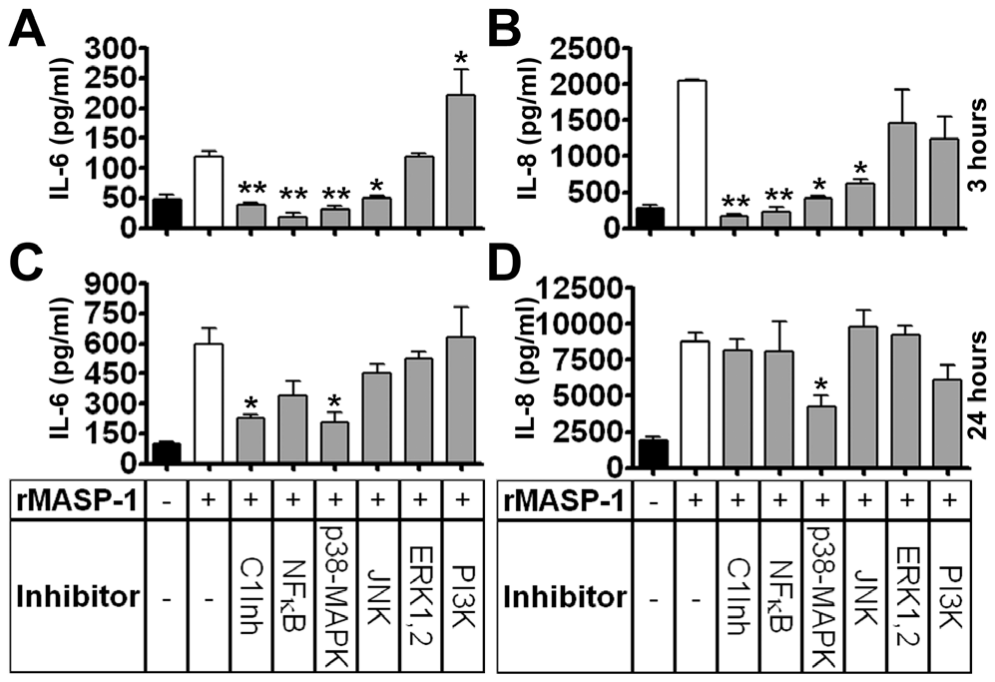
The signaling pathways of IL-6 and IL-8 production induced by rMASP-1. The cells were pre-incubated with signaling pathway inhibitors for 30 minutes and then treated with 2 µM rMASP-1 for 3 (**A, B**) or 24 (**C, D**) hours. Supernatants were collected and analyzed with commercial IL-6 (**A, C**) and IL-8 (**B, D**) ELISA kits. C1-Inhibitor (C1Inh) was applied differently; it was premixed with rMASP-1 for 30 minutes and then, the mixture was added to the cells. Each panel was calculated as the mean (+/−SEM) of 3 independent experiments using HUVECs from different donors. The significance of the differences among rMASP-1 and other treatments is shown. *: p<0.05, **: p<0.01.

Cytokine production can be triggered through various signaling pathways, most of which can be activated also by rMASP-1. Thus, we used several commercially available signaling pathway inhibitors to identify the most important pathways required for IL-6 and IL-8 production. HUVECs had been pre-incubated with the inhibitors for 30 minutes and then, treated with 2 µM rMASP-1 for 3 or 24 hours. The expression of both IL-6 and IL-8 could be inhibited by NFkappaB, p38-MAPK, or JNK at 3 hours of rMASP-1 activation (Figure 5/A,B), whereas only the p38-MAPK inhibitor was able to block the production of both cytokines significantly, at 24 hours (Figure 5/C,D). ERK 1/2 and PI3-kinase inhibitors had no blocking effect at any time. PI3-kinase inhibitor, however, increased the IL-6 production induced by rMASP-1 at 3 hours. Furthermore, C1-Inhibitor blocked the expression of both cytokines when it was pre-incubated with rMASP-1 for 30 minutes before adding to the cells. Interestingly, while the blocking effect of the C1-Inhibitor on IL-6 persisted for 24 hours, it had no effect on IL-8 production when applied for 24 hours. Theoretically, this could happen if the rMASP-1/C1-Inhibitor complex disintegrates within a few hours (unlike C1-Inhibitor complexed with C1r/s, which is very stable), and rMASP-1 regains its activity. To test this hypothesis, we explored the disintegration of the rMASP-1/C1-Inhibitor complex. rMASP-1 was incubated with or without equimolar amounts of C1-Inhibitor for 24 hours, and small aliquots were tested with a fluorometric substrate-cleavage assay at different times. We found that rMASP-1 enzymatic activity decreased only slightly during the 24-hour incubation period (initial reaction rates of 20 nM rMASP-1 at 3000 nM substrate concentration: 2314 ± 110 pM substrate/sec to 1863 ± 30 pM substrate/sec). The proteolytic effect of the rMASP-1/C1-Inhibitor complex remained under the detection limit during the entire experiment.

### Activation of neutrophil granulocytes by the supernatant of rMASP-1 treated HUVECs

**Figure 6:**
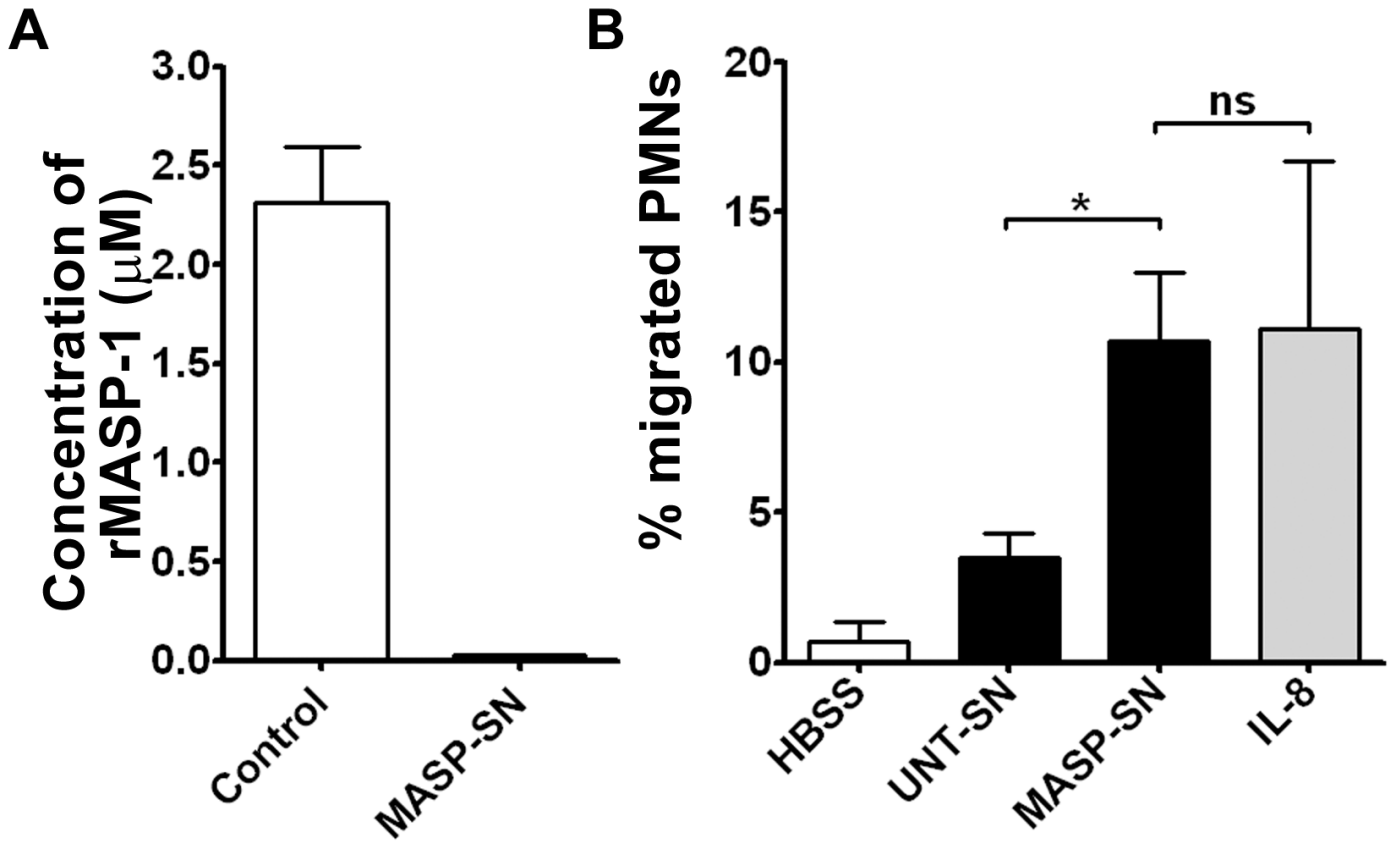
The migration of PMNs activated by the rMASP-1-treated HUVEC supernatant. HUVEC cells were treated with 2 µM rMASP-1, or left untreated for 30 minutes. Then, the medium was changed to rMASP-1 free HBSS for 2.5 hours and then MASP-SN and UNT-SN were collected. Residual rMASP-1 concentration of MASP-SN was checked by LPAPR-AMC fluorescent substrate assay, where the Control column shows the rMASP-1 concentration of the rMASP-1 treated HUVEC supernatant before changed to HBSS (**A**). PMNs were isolated from venous blood collected from 2 healthy volunteers. 10?5 cells were seeded in 3-µm pore size transwell inserts and placed for an hour into the wells containing the MASP-SN, UNT-SN or HBSS with/without 2 ng/ml IL-8. The percentage of transmigrated/total cells was calculated as the mean (+/−SEM) of 3 different chemotaxis assays (**B**). The significance of the differences among rMASP-1 and other treatments is shown. *: p<0.05, ns: non-significant.

Since the advent of high-sensitivity of ELISA assays, the biological significance of cytokine production can be overestimated by the statistical methods. Thus, we tested whether the short-term activation of HUVECs with rMASP-1 was able to induce cytokines in an amount sufficient to stimulate neutrophil granulocytes (PMN). To assess the quick response of PMNs, we monitored superoxide (O_2_^−^) production, and performed chemotactic measurements. To avoid the direct effect of rMASP-1 on PMNs, media with or without rMASP-1 were replaced after 30 minutes and further incubated in fresh, rMASP-1 free HBSS for additional 2,5 hours before collection (MASP-SN or UNT-SN, respectively), as it was described in *Materials and Methods*, and we verified that the MASP-SN was rMASP-1 free (Figure 6/A). First, we assessed superoxide production over 20 minutes by lucigenin-based chemiluminescence. We found that both supernatants induced extremely weak O_2_^−^ production, and MASP-SN did not differ from UNT-SN (1.35% vs. 1.55%, *p* = 0.31, where the maximal superoxide production, generated by 100 µM PMA, was regarded as 100%). PMNs were also preconditioned with MASP-SN or UNT-SN for 20 minutes and then, stimulated with PMA or fMLP. MASP-SN priming had no effect on the O_2_^−^ generation of PMNs compared to UNT-SN using either stimulator (15.52% vs. 15.96% for fMLP, *p* = 0.68 and 70.42% vs. 71.13% for PMA, *p* = 0.76).

Next, we performed a chemotactic assay, where PMNs were pipetted into transwell inserts and placed into 24-well plates loaded with HBSS (with Ca^2+^/Mg^2+^, as a negative control), MASP-SN, UNT-SN, or IL-8 (2 ng/mL, as a positive control). Compared with HBSS, even the untreated HUVEC supernatant induced chemotaxis, and this was further increased by MASP-SN. The chemotactic effect of MASP-SN was similar to that of 2 ng/mL IL-8 (Figure 6/B).

## Discussion

We found that rMASP-1 treatment activated JNK and CREB phosphorylation. The most important pro-inflammatory cytokines including IL-1beta and TNFalpha can simultaneously activate different MAPKs and NFkappaB. MASP-1 and these three pro-inflammatory activators appear to use similar pathways; however, in case of MASP-1, the MAPK pathways may be more effective than NFkappaB signaling. CREB, another important transcription factor of pro-inflammatory cytokine production [9], [18], [25], can be phosphorylated and activated via two main routes: by cAMP activated protein kinase A, and by p38-MAPK [9], [26]. Since we have found previously that p38-MAPK pathway is readily activated by rMASP-1, the phosphorylation of CREB is not unexpected [6]. Thrombin, a serine protease similar to MASP-1 [27], also induces CREB phosphorylation, which supports that PAR signaling can lead to the activation of the CREB transcription factor [28].

The balance between the pro- and anti-inflammatory phenotypes is partially dependent on the cytokine pattern produced by endothelial cells. The pro-inflammatory cytokines IL-1alpha, IL-6, IL-8, MCP-1 and TNFalpha are representative of the endothelial phenotypic changes occurring during inflammation [29]. To counterbalance the effect of pro-inflammatory cytokines, the endothelium can produce anti-inflammatory cytokines, e.g. IL-1receptor antagonist, which can downregulate the inflammatory processes [30]. First, we screened the cytokine pattern of endothelial cells in response to rMASP-1 by bead-array and qPCR, to determine the most important targets for future studies. rMASP-1-triggered endothelial cells produced IL-6 and IL-8 at mRNA and protein levels, which switched the endothelial cells to a pro-inflammatory phenotype.

We described that the rMASP-1-induced production of the IL-6 and IL-8 cytokines was dose dependent. Interestingly, although we showed that rMASP-1 is able to increase the expression of IL-1ra, MCP-1 and TNFalpha at mRNA level, we could not detect any dose-dependent production of these chemokines at protein level. Increased mRNA levels without protein secretion predicts that MASP-1 may act in synergy with pro-inflammatory factors to produce IL-1ra, MCP-1 and TNFalpha. MASP-1 is one of the most abundant proteases in the plasma; its mean systemic concentration is 11 µg/ml [31] (143 nM). We found that the measurable effect of rMASP-1 on endothelial cytokine production was as low as 250 nM. However, at the site of complement activation and microvessel injury, local MASP-1 concentration may greatly exceed 143 nM. In these *in vivo* physiological/pathophysiological conditions, therefore, MASP-1 concentrations may be similar to those tested in our *in vitro* experiments.

One-by-one comparisons of the production of cytokines may be misleading and useless, because the *in vivo* concentrations of endothelial cell activators are different. An alternative approach is to evaluate complex cytokine patterns [8], focusing on the relative proportions of the production of individual cytokines. The three most-cited factors acting as inflammatory signals and triggering cytokine production by endothelial cells are LPS, TNFalpha, and IL-1beta [29]. Compared with these, rMASP-1 has a lesser overall effect on cytokine production, and no effect whatsoever on MCP-1 production, whereas it induces significant IL-6 and IL-8 secretion. In contrast, LPS, TNFalpha, and IL-1beta stimulate IL-6, IL-8, and MCP-1 production, although to different extents [8]. Histamine, bradykinin, and thrombin are also well-described endothelial activators with pro-inflammatory effects, and additional actions that are not related to the inflammatory process [32]. Histamine and thrombin induced weak IL-6 and IL-8 expression, and left MCP-1 expression unchanged (data not shown). Thus, as regards the cytokine pattern, rMASP-1 is more similar to these two factors than to LPS, IL-1beta, and TNFalpha, or bradykinin.

Monocyte/granulocyte recruitment occurs at the site of inflammation within minutes, and the prompt release of relevant mediators from the endothelium underlies this rapid mechanism [33]. To control the migration of different cell subsets, the endothelial cells store granules of different cytokine composition [34]. MCP-1 and IL-8, the two well-characterized chemoattractants for monocytes and neutrophil granulocytes, are stored separately, and excreted in different quantities and with dissimilar velocities [34], [35]. The rapid, thrombin-induced secretion of IL-8 takes place without *de novo* protein synthesis, while the pro-inflammatory, endothelial activators such as TNFalpha and IL-1beta cause consistent and prolonged IL-8 production [15], [33], [35]. IL-6 may co-localize with IL-8, but their separate storage has also been described [15]. We found that rMASP-1 and TNFalpha induced similar IL-6 excretion. However, rMASP-1 evoked much faster release of IL-8 than TNFalpha, and this implicates an instant chemotactic role for MASP-1-induced endothelial cells in the early immune response.

To better understand the secretion of cytokines, we tried to identify the signaling pathways involved in MASP-1-induced IL-6 and IL-8 production. The p38-MAPK inhibitor could abolish IL-6 and IL-8 secretion at both 3 and 24 hours, whereas the NFkappaB and JNK inhibitors were effective only at 3 hours. The ineffectiveness of JNK and NFkappaB inhibitors at 24 hours could be explained by the degradation of pathway inhibitors. However, Cherla et al. showed that these inhibitors were able to block LPS-induced IL-8 production completely at 24 hours [36]. It implicates that IL-6/IL-8-containing granules may translocate to Weibel-Palade bodies (WPBs) and subsequently undergo exocytosis in response to rMASP-1, as it has been described in case of IL-1beta [37], and this translocation as well as the process of exocytosis may be triggered via several pathways [38]. The p38-MAPK pathway, by contrast, appears to have a major role in the *de novo* synthesis of IL-6 and IL-8, which become the predominant cytokines after 24 hours. These cytokines carry several transcription factor-binding sites at their promoter region, of which NFkappaB, AP-1 and CREB they share in common. The MCP-1 promoter region also has binding sites for NFkappaB and AP-1, but not for CREB [16]. Since rMASP-1 did not induce MCP-1 expression, the difference in the presence of CREB binding sites between MCP-1 and IL-6/IL-8 promoters may emphasize the importance of CREB activation in rMASP-1 induced IL-6 and IL-8 production. PI3-kinase has only a weak effect on rMASP-1 induced IL-6 secretion at 3 hours. It is not surprising that this effect was an increased cytokine production instead of inhibition because Kim et al. described that PI3-kinase can suppress the NFkappaB pathway in HUVECs [39] and we showed that NFkappaB plays a role in the early IL-6 production by rMASP-1. Unexpectedly, although the C1-Inhibitor partially blocked IL-6 production at 24 hours, it had no effect on IL-8 secretion at this time. Since we showed that the rMASP-1/C1-Inhibitor complex did not disintegrate within 24 hours, an alternative mechanism should be presumed. As the C1-Inhibitor complex – but not the active C1-Inhibitor – binds to low-density lipoprotein receptor-related proteins (LRPs) [40] expressed on endothelial cells [41], it appears plausible that the rMASP-1/C1-Inhibitor complex may induce IL-8 production via LRPs after 24 hours of activation.

The differential expression of IL-6/IL-8 and of MCP-1 prompted us to assume that MASP-1-induced endothelial cells preferentially influence neutrophil functions. Neutrophil granulocytes are the first line of cellular defense against bacterial and fungal pathogens. Their activation begins within the blood vessels, whereby chemotaxis is induced by several pro-inflammatory chemoattractants, including IL-8, fMLP, and C5a [42]. This rapid process is followed by transmigration, further activation then phagocytosis and ROS generation. The rMASP-1-induced HUVEC supernatant did not trigger or prime O_2_^−^ production, whereas it had a significant effect on the rapid chemotaxis of neutrophils. Moreover, this effect was similar to that of 2 ng/mL IL-8, which is nearly the same dose measured in the rMASP-1-treated HUVEC supernatant.

In our study, we assessed the effects of rMASP-1 on HUVECs as the most widely used endothelial cell model system. However, further experiments are required to compare MASP-1 responsiveness of endothelial cells with different origin.

MASP-1, a crucial element of the complement lectin pathway [2], is immediately activated upon microbial infection, enabling the downstream mediators of the complement system to opsonize and/or kill microbes. Our findings implicate that rMASP-1 can promptly attract neutrophil granulocytes by activating endothelial cells. This action may link the complement lectin pathway to targeted neutrophil activation, as two highly effective mechanisms of innate antimicrobial immunity.
